# Dynamin-2 Regulates Fusion Pore Expansion and Quantal Release through a Mechanism that Involves Actin Dynamics in Neuroendocrine Chromaffin Cells

**DOI:** 10.1371/journal.pone.0070638

**Published:** 2013-08-05

**Authors:** Arlek M. González-Jamett, Fanny Momboisse, María José Guerra, Stéphane Ory, Ximena Báez-Matus, Natalia Barraza, Valerie Calco, Sébastien Houy, Eduardo Couve, Alan Neely, Agustín D. Martínez, Stéphane Gasman, Ana M. Cárdenas

**Affiliations:** 1 Centro Interdisciplinario de Neurociencia de Valparaíso, Facultad de Ciencias, Universidad de Valparaíso, Gran Bretaña, Playa Ancha, Valparaíso, Chile; 2 Institut des Neurosciences Cellulaires et Intégratives, Centre National de la Recherche Scientifique (CNRS UPR 3212), and Université de Strasbourg, Strasbourg, France; 3 Departamento de Biololgía, Facultad de Ciencias, Universidad de Valparaíso, Gran Bretaña, Playa Ancha, Valparaíso, Chile; The University of Queensland, Australia

## Abstract

Over the past years, dynamin has been implicated in tuning the amount and nature of transmitter released during exocytosis. However, the mechanism involved remains poorly understood. Here, using bovine adrenal chromaffin cells, we investigated whether this mechanism rely on dynamin’s ability to remodel actin cytoskeleton. According to this idea, inhibition of dynamin GTPase activity suppressed the calcium-dependent *de novo* cortical actin and altered the cortical actin network. Similarly, expression of a small interfering RNA directed against dynamin-2, an isoform highly expressed in chromaffin cells, changed the cortical actin network pattern. Disruption of dynamin-2 function, as well as the pharmacological inhibition of actin polymerization with cytochalasine-D, slowed down fusion pore expansion and increased the quantal size of individual exocytotic events. The effects of cytochalasine-D and dynamin-2 disruption were not additive indicating that dynamin-2 and F-actin regulate the late steps of exocytosis by a common mechanism. Together our data support a model in which dynamin-2 directs actin polymerization at the exocytosis site where both, in concert, adjust the hormone quantal release to efficiently respond to physiological demands.

## Introduction

Dynamin is a mechano-GTPase encoded by three distinct genes (DNM1, DNM2 and DNM3) that generates membrane deformation and triggers membrane fission [1]. Its best characterized function is the scission of nascent vesicles from the plasma membrane during endocytosis. Of the three dynamin isoforms only dynamin-2 is ubiquitously expressed, while dynamin-1 is exclusively expressed in neuronal tissue, and dynamin-3 is only present in brain, testis, heart and lungs [2], [3]. Studies in knock-out animals show that only dynamin-2 is critical during early embryonic development [4] and that the absence of dynamin-1 or -3 can be compensated by the other isoforms [5]. From these findings arises the idea that the different dynamin isoforms have overlapping roles and their relative contribution to endocytosis in a given tissue is mostly determined by their abundance rather than on structural specialization [5].

Dynamin participates in several cellular processes that are dependent on the actin cytoskeleton dynamics, some of them are actin comet [6], [7] lamellipodia formation [8], T cell activation [9], phagocytosis [10] and different types of endocytosis [11]–[14]. Furthermore, a functional link between dynamin and actin has been observed during endocytosis, where one regulates the recruitment of the other [14] and both work synergistically to efficiently catalyze membrane scission [12]. The exact mechanism of the crosstalk between dynamin and actin is not completely clear, but some evidences suggest that dynamin binds directly to actin filaments to promote its polymerization by displacing the actin capping protein gelsolin [15]. Additionally, dynamin can control the stability of actin filaments in association with the actin-binding protein cortactin, in a GTP hydrolysis-dependent way [16], [17].

In neuroendocrine cells, both actin and dynamin have been involved in the regulation of the exocytotic process. On one hand, cortical actin network is reorganized during exocytosis [18], wherein it regulates the expansion of the fusion pore [19], an intermediate structure formed during the fusion of the secretory vesicle with the plasma membrane [20]. On the other hand, dynamin appears to regulate both fusion pore expansion [21] and closure [22], [23], and to control the quantal size of release events [24]–[27]. These actions have been attributed to the neuronal isoform dynamin-1 [21], [23], [25], while dynamin-2 has been proposed to specialize in the regulation of compensatory endocytosis [28]. This functional divergence suggests that different dynamin isoforms are specifically tuned to regulate different stages of the granule life cycle rather than having overlapping roles.

Here using molecular tools that disrupt endogenous dynamin-2 function or expression we demonstrate that this isoform, which is highly expressed in bovine chromaffin cells (BCC), controls fusion pore expansion and quantal size. Additionally, we found that dynamin-2 regulates the organization and Ca^2+^-dependent assembly of cortical actin. Similarly to that observed with the disruption of dynamin-2 function or expression, disturbance of actin dynamics increased fusion pore duration and quantal size, but these effects were no longer visible when endogenous dynamin-2 was already disrupted. These results strongly suggest that dynamin-2 regulates different stages of exocytosis through a mechanism that involves actin dynamics.

## Results

### Dynamin-2 is Highly Expressed in Bovine Chromaffin Cells

**Figure 1:**
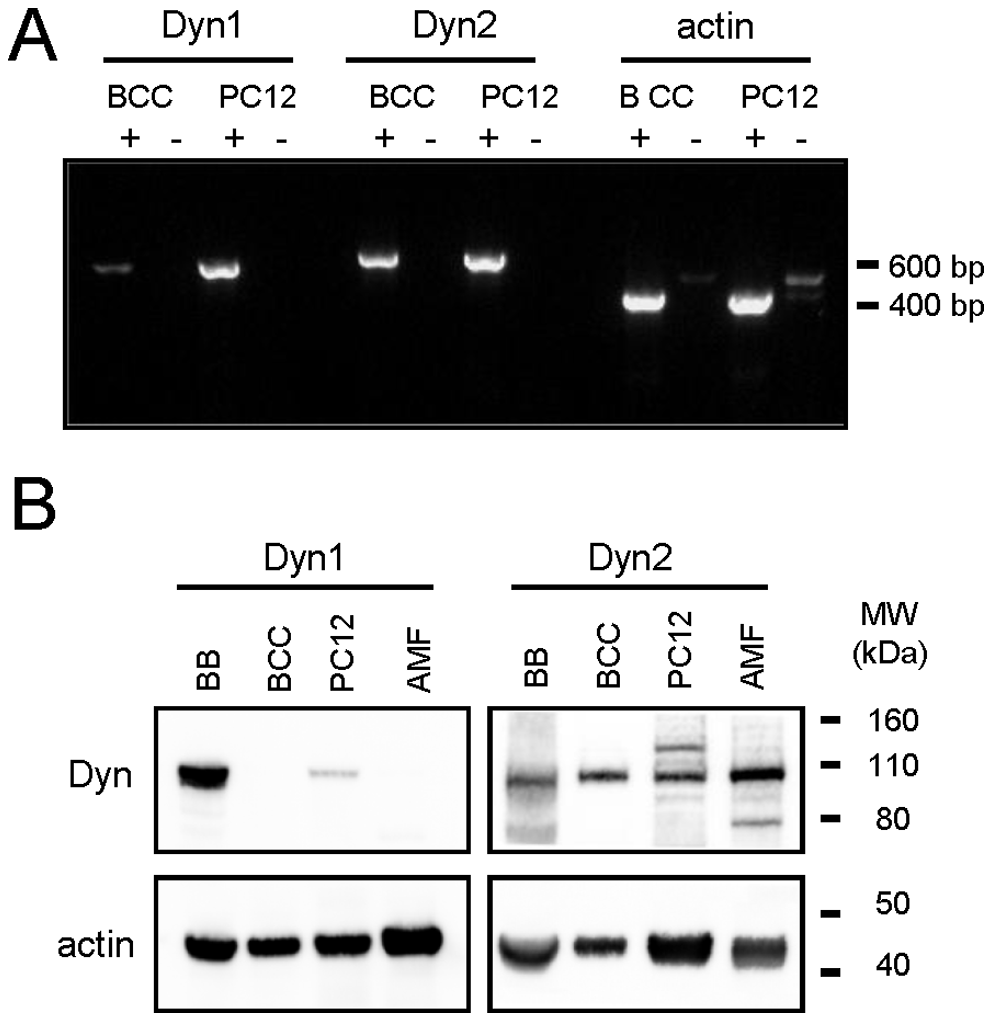
Dynamin-2 is highly expressed in bovine adrenal chromaffin cells. Relative expression of dynamin-1 and -2 was evaluated in BCC and PC12 cells using RT-PCR and western-blot. ***A:*** RT-PCR results. Note that in BCC, the amount of dynamin-1 mRNA is significantly lower as compared with the dynamin-2 transcript. Actin was used as an amplification control. A control without reverse transcriptase (RT) was performed in parallel to rule out genomic contamination. Signs+or – indicate, respectively, the presence or absence of RT during amplification. **B:** Western-blot results. Proteins from bovine brain (BB), BCC, PC12 cells and primary adult mouse fibroblasts (AMF) extracts were separated by electrophoresis on a 4–12% polyacrylamide gel gradient and analyzed by western blot. Note that dynamin-1 level was almost undetectable in BCC. β-actin was used as loaded control.

To evaluate the relative expression of dynamin isoforms 1 and 2 in bovine chromaffin cells (BCC), we performed RT-PCR and western blot assays. RT-PCR showed that both isoforms are expressed, but dynamin-1 mRNA levels are substantially lower in BCC compared to the transcript coding for dynamin-2 (Fig. 1A). At protein level, western blot analysis with an antibody that is specific for dynamin-1 revealed a 100 kDa band in the bovine brain extract, but failed to detect any protein in BCC (Fig. 1B). In contrast, western blotting with an antibody raised against dynamin-2 allowed us to detect a robust 100 kDa band in BCC as well as in the bovine brain and mouse fibroblast. As these results indicate that dynamin-2 is highly expressed in BCC, we investigated the role of this isoform in the exocytosis.

### Endogenous Dynamin-2 Controls the Quantal Release of Catecholamines in Chromaffin Cells

To study the role of endogenous dynamin-2 in exocytosis we used a small interfering RNA strategy (iRNADyn2) to down-regulate its expression [29] or a dominant negative mutant (Dyn2K44A) to inhibit its GTPase activity [30], [31]. The efficiency of these constructs in BCC was evaluated using an anti-dopamine-beta-hydroxylase (DBH) antibody internalization assay to monitor compensatory endocytosis of chromaffin granules [32], a process known to be dependent on dynamin-2 [28]. iRNADyn2 significantly reduced the DBH internalization by 53% compared to cells expressing the empty vector pEGFP or an unrelated iRNA (iRNA-UnR) (Fig. S1). Similarly, Dyn2K44A expression reduced the DBH internalization by 52% compared to the expression of wild-type dynamin-2 (Dyn2WT); thus, the functional efficacy of these constructs was confirmed.

**Figure 2:**
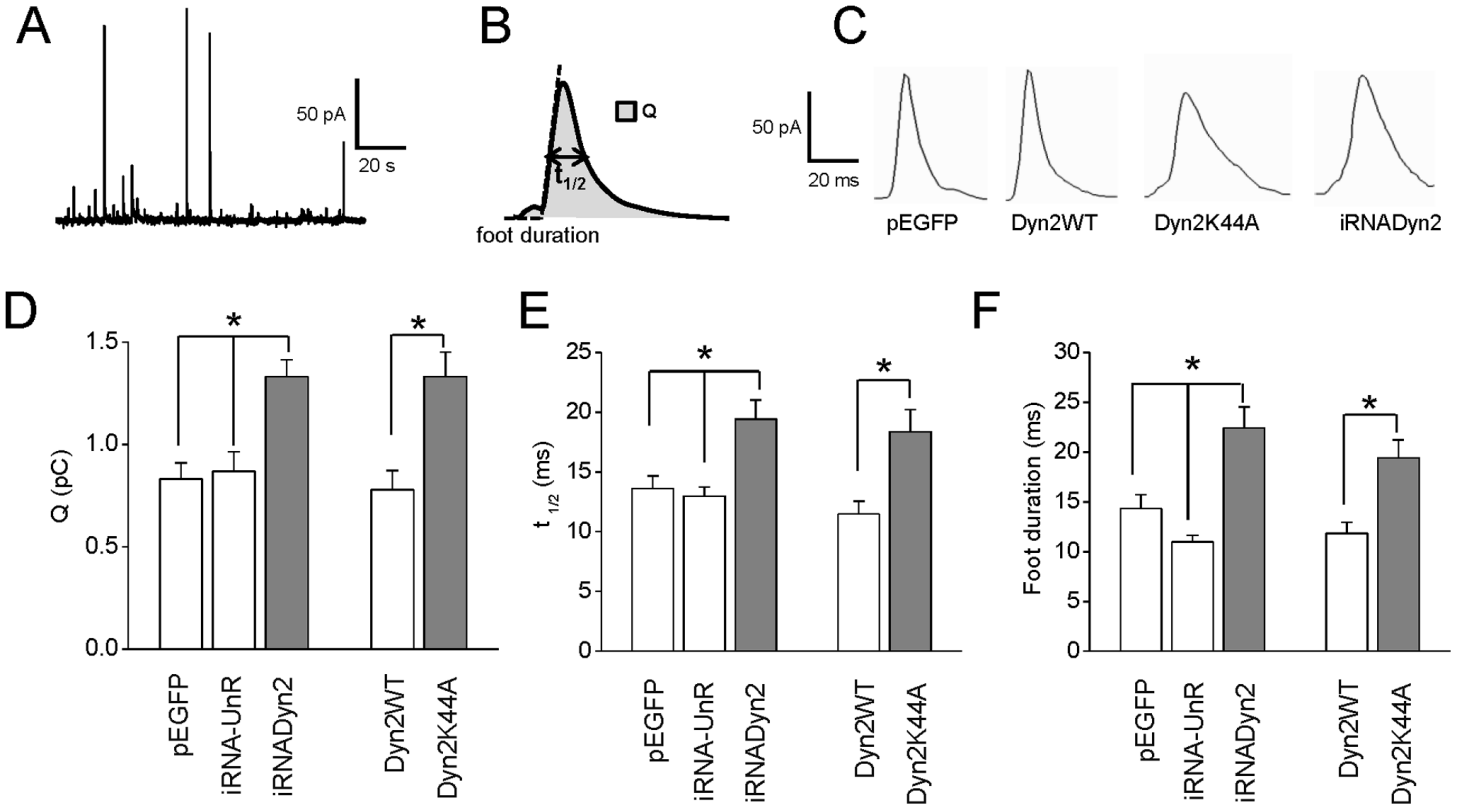
Endogenous dynamin-2 regulates the quantal release and expansion of the fusion pore. Exocytosis was evoked with 10 µM of the nicotinic agonist DMPP and monitored using amperometry. **A**: A typical amperometry trace in a single control chromaffin cell stimulated with 10 µM DMPP. **B:** Scheme of an amperometric spike with the analyzed parameters: quantal size (Q), half width (t_1/2_) and foot duration. **C**: Typical amperometric spikes induced by 10 µM DMPP in cells transfected with pEGFP, Dyn2WT, Dyn2K44A or iRNADyn2. **D–F**: Data show average values ± SEM of Q (C), t_1/2_ (D) and foot duration (E) of amperometric events in cells transfected with pEGFP (n = 35), iRNA-UnR (n = 28), Dyn2WT (n = 15) in white bars, and cells transfected with iRNADyn2 (n = 29) or Dyn2K44A (n = 18) in gray bars. All amperometric parameter values correspond to the median values of the events from individual cells, which were subsequently averaged per treatment group. Thus, n corresponds to the number of cells in each treatment group. Note that the down-regulation of dynamin-2 (iRNADyn2) or the inhibition of its GTP-ase activity (Dyn2K44A) significantly increased Q, t_1/2_ and foot duration of the exocytotic events evoked by 10 µM DMPP. *p<0.05 (Kruskal-Wallis test).

Then, we analyzed the contribution of dynamin-2 to calcium-regulated exocytosis. Catecholamine release from adrenal chromaffin cells was stimulated with the nicotinic agonist 1,1-dimethyl-4-phenyl-piperazinium (DMPP) to mimic the physiological condition. The different stages of exocytosis were monitored by amperometry. From each amperometric spike we analyzed the quantal size (Q), which is proportional to the amount of catecholamines released per event, and the half-width (t_1/2_) that reflects the duration of the exocytotic events (Fig. 2A). Figure 2B shows examples of amperometric spikes induced by 10 µM DMPP in cells expressing pEGFP, Dyn2WT, Dyn2K44A and iRNADyn2, respectively. Stimulation of untransfected BCC with a 10 s pulse of 10 µM DMPP produced 20.1±2.2 amperometric spikes in 100 s (n = 45), which mean Q and t_1/2_ values were 0.8±0.1 pC and 14.2±0.8 ms, respectively. The expression of iRNAUnR, Dyn2WT or pEGFP vectors did not change significantly any of the amperometric parameters (Fig. 2C–D and Table 1). Conversely, the disruption of endogenous dynamin-2 expression by iRNADyn2 significantly increased Q (1.3±0.1 pC) and t_1/2_ (19.4±1.6 ms) without significant differences in the number of exocytotic events (23.0±3.2) as compared with those obtained in pEGFP or iRNAUnR transfected cells. Likewise, the expression of Dyn2K44A significantly increased Q (1.3±0.1 pC) and t_1/2_ (18.4±1.9 ms) without affecting the number of amperometric spikes (21.5±3.1) (Fig. 2C–D and Table 1). Finally, the expression of the different constructs did not affect the amplitude of the amperometric spikes (see Table 1). These data show that, under stimulation of nicotinic receptors, dynamin-2 adjusts the amount of hormones released via it GTPase activity.

**Table 1:** Amperometric parameters of exocytotic events induced by 10 µM DMPP in cells transfected with pEGFP, UnR-iRNA, iRNADyn2, Dyn2WT or Dyn2K44A.

	pEGFP	UnR-iRNA	iRNADyn2	Dyn2WT	Dyn2K44A
Number of events	17.7±1.7	24.2±3.6	23.0±3.2	20.2±2.4	21.5±3.1
Q (pC)	0.83±0.08	0.87±0.09	1.33±0.08*	0.78±0.09	1.33±0.12†
t_1/2_ (ms)	13.6±1.1	13.0±0.8	19.4±1.6*	11.5±1.0	18.4±1.8†
Imax (pA)	51.5±3.8	55.1±5.1	59.4±5.6	65.3±7.9	62.4±3.7
Foot frequency(%)	63.3±5.5	57.9±3.7	57.8±3.6	61.6±10	63.4±8.3
Foot amplitude(pA)	12.4±1.3	13.6±1.0	11.3±0.9	11.6±1.1	12.1±1.6
Foot duration(ms)	14.3±1.4	11.0±0.6	22.4±2.1*	11.8±1.1	19.4±1.8†
Number ofcells	35	28	29	15	18

Data are means ± SEM of averages, where n is the number of cells. Imax corresponds to the spike amplitude.

* p<0.05 compared with pEGFP-transfected cells;

† p<0.05 compared with Dyn2 WT-transfected cells (Kruskal-Wallis test).

### Endogenous Dynamin-2 Controls the Expansion of the Initial Fusion Pore

To investigate whether dynamin-2 regulates the dynamics of the fusion pore, we analyzed the small pre-spike current, usually called “foot”, which reflects the slow release of transmitters through an initial fusion pore [33]. The “foot” duration correlates with the stability of the fusion pore [20], while its amplitude is proportional to its conductance [34]. In untransfected cells, 60.6±4.3% of amperometric spikes exhibited foot signals with mean duration of 14.6±1.6 ms and mean amplitude of 12.9±1.1 pA. Transfection with pEGFP, iRNA-UnR or Dyn2WT did not significantly change these values (see values in Table 1). Interestingly, disruption of endogenous dynamin-2 by expression of iRNADyn2 or inhibition of its GTPase activity by overexpressing Dyn2K44A prolonged the duration of the foot signals (22.4±2.1 and 19.4±1.8 and ms, respectively), as compared with pEGFP- or Dyn2WT-transfected cells (14.3±1.4 and 11.8±1.1 ms, respectively) (Fig. 2E), but it did not have any significant effect on foot frequency or foot amplitude of the exocytotic events triggered by 10 µM DMPP (see values in Table 1). These results indicate that endogenous dynamin-2 controls the fusion pore expansion, without influencing its conductance.

### Dynamin-2 Controls Cortical Actin Assembly in Bovine Chromaffin Cells

**Figure 3:**
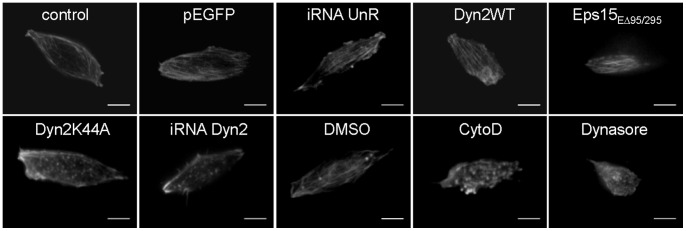
Impaired function or expression of dynamin-2 change F-actin organization pattern. Cells were transfected with Life-act-ruby (n = 11) or co-transfected with Life-act-ruby and pEGFP (n = 34), iRNA-UnR (n = 9) Dyn2WT (n = 21), Dyn2K44A (n = 31), iRNADyn2 (n = 38) or Eps15ED95/295 (n = 17) plasmids and visualized by TIRF microscopy 48 h later. To evaluate the effects of a pharmacological inhibition of dynamin, cells transfected with Life-act-ruby were treated with 100 µM dynasore (n = 28), or the vehicle DMSO (n = 25) during 1 hr at 37°C. The 81.8% of control cells exhibited a “normal” pattern with clear cortical actin fibers. This value was not significantly different in cells expressing pEGFP (73.6%), iRNA-UnR (88.9%) or Dyn2WT (85.7%) constructs. However, the expression of Dyn2K44A or iRNADyn2, as well as the treatment with dynasore, modified the cortical actin organization and 80.6%, 92.1% and 71.4% of the cells, respectively, exhibited a “punctuate” pattern. The treatment with 4 µM CytoD during 10 minutes at 37°C produced exactly the same effect: 84.6% of the cells displayed a “punctuate” pattern. Eps15ED95/295 expression did not alter actin organization (82.4% of cells exhibited a normal pattern), indicating that dynamin, but not of endocytosis disruption, modified the actin cytoskeleton pattern. Scale bar = 5 µm.

To explore the possibility that dynamin-2 actions on the exocytosis were linked to dynamin’s ability to remodel actin cytoskeleton, we first evaluated the effect of dynamin disruption on the actin organization. In order to analyze the cortical actin distribution in BCC we visualized actin filaments (F-actin) at the plasma membrane level using total internal reflection fluorescence microscopy (TIRFM) in living cells transfected with the fluorescent dye *Life-act-ruby*. *Life-act-ruby* is a peptide that binds F-actin with low affinity and thus constitutes a powerfull tool to visualize actin organization in vivo without affecting its polymerization/depolymerization dynamics [35]. Under these experimental conditions, cortical actin was observed as a network of fibers of different thickness. This pattern was maintained in cells expressing pEGFP, iRNAUnR or Dyn2WT (Fig. 3). However, the expression of iRNADyn2 or Dyn2K44A, as well as the inhibition of dynamin GTPase activity with dynasore, modified this pattern displaying a punctuated actin staining, similar to that observed in the presence of the actin-disrupting drug cytochalasine D (CytoD) (Fig. 3). These effects were unrelated to the inhibition of endocytosis since the expression of Eps15EΔ95/295, a mutant of the epidermal growth factor receptor substrate 15 (Eps15) that admittedly inhibits compensatory endocytosis in chromaffin cells [32], had no effect on the actin cytoskeleton organization (Fig. 3).

**Figure 4:**
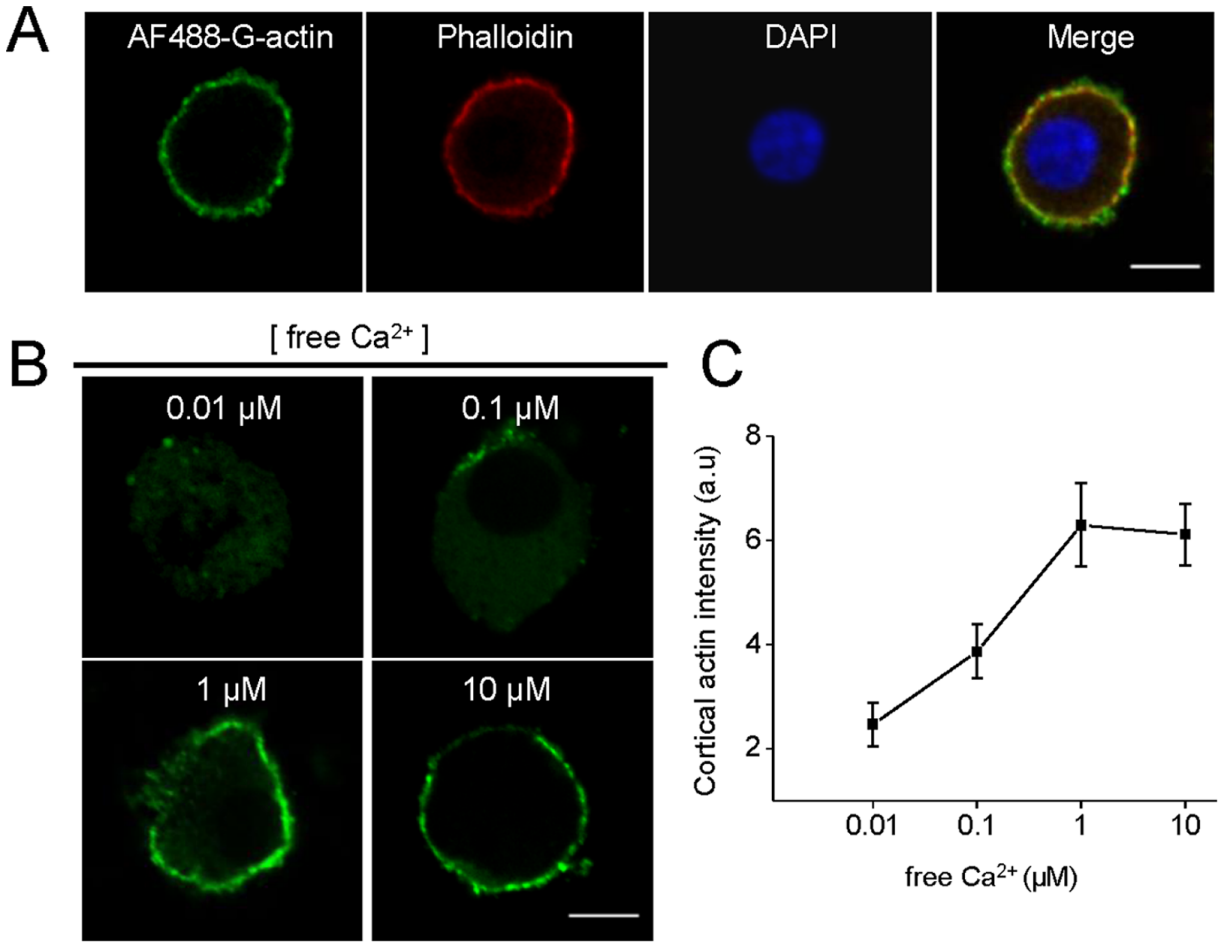
Calcium-dependent cortical actin polymerization in permeabilized chromaffin cells. Cultured chromaffin cells were permeabilized in buffer KGEP (mM: 139 K^+^-glutamate, 20 Pipes, 5 EGTA, 2 ATP-Mg and 0.01 free calcium, pH 6.6) during 6 minutes with 20 µM digitonin in the presence of 0.3 µM Alexa-Fluor488-G-actin conjugate (AF488-G-actin), fixed and visualized by confocal microscopy. **A:** Total F-actin was stained using 1 µM phalloidin-rodhamine B (red) and nuclei were stained with 5 µg/ml DAPI (blue). Note that newly synthesized actin was incorporated into pre-existing cortical filaments. **B–C:** The new formation of cortical actin filaments was assessed by quantifying AF488-G-actin staining mean intensity at the cell periphery in the presence of increasing free Ca^2+^ concentrations. Note that maximal cortical actin polymerization was observed at a range of 1–10 µM of free Ca^2+.^ Scale = 10 µm. Data are means of cortical actin fluorescence intensity from at least 12 cells per each Ca^2+^ concentration (12 cells for 0.01 µM Ca^2+^, 13 cells for 0.1 µM Ca^2+^, 15 cells for 1 µM Ca^2+^,and 18 cells for 10 µM Ca^2+^).

**Figure 5:**
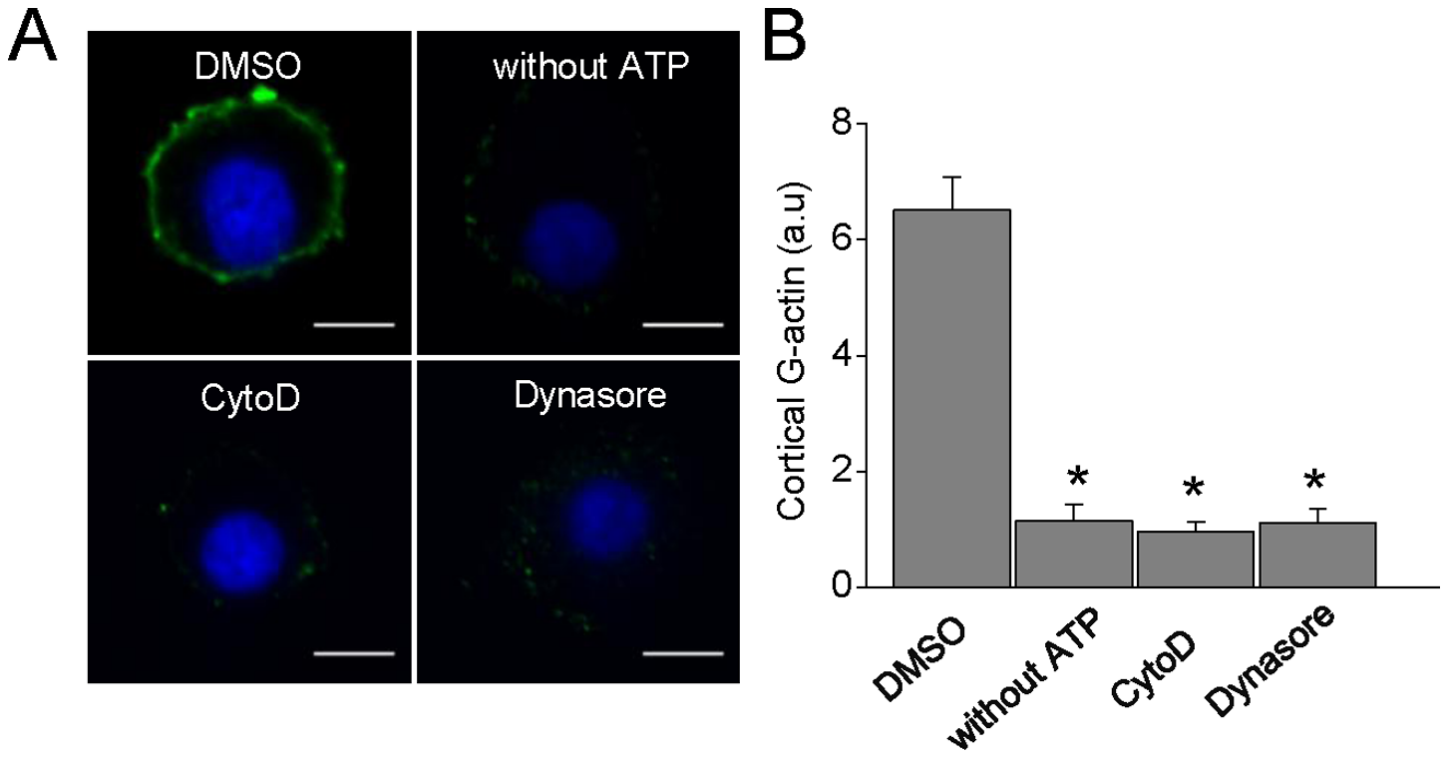
Inhibition of dynamin GTP-ase activity suppresses Ca^2+^-dependent *de novo* cortical actin polymerization. **A:** Representative images of F-actin formation in cells permeabilized in the presence of 10 µM free Ca^2+^. Note that no new polymerized cortical actin was observed when the permeabilization was performed in the absence of ATP-Mg (n = 16) or in the presence of 4 µM CytoD (n = 27) or 100 µM dynasore (n = 28) Scale bar = 10 µm **B:** Quantification of G-actin staining mean intensity at the cell periphery. Data are means of cortical actin fluorescence intensity *p<0.05 compared with cells treated with DMSO (ANOVA).

Previously, we proposed a *de novo* formation of actin filaments in the course of exocytosis in chromaffin cells [18], [36]. Therefore, we next evaluated the effects of the pharmacological inhibition of dynamin GTPase activity on *de novo* actin filament formation in permeabilized cells. As shown in Figure 4A G-actin polymerized into the pre-existing cortical F-actin, forming a ring beneath the plasma membrane. The new formation of cortical actin filaments was critically dependent on the Ca^2+^ levels, being maximal at a range of 1 to 10 µM free Ca^2+^ (Fig. 4B–C), which is the range of Ca^2+^ concentration known to favor exocytosis in permeabilized chromaffin cells [37]. Interestingly, this Ca^2+^-dependent cortical actin polymerization was abolished in the presence of the inhibitor of dynamin GTPase activity dynasore, alike to that observed in the presence of CytoD or in the absence of ATP (Fig. 5A–B).

Overall these data suggest that the GTP-ase activity of dynamin-2 is required for the proper cortical actin organization during exocytosis in bovine chromaffin cells.

### Actin Dynamics Regulates the Late Stages of Exocytosis

In order to analyze how the disruption of actin polymerization affects the exocytosis, we evaluated the effect of CytoD on the amperometric parameters. Similarly to that observed with iRNADyn2 and Dyn2K44A, CytoD treatment significantly increased Q (1.62±0.16 pC) and t_1/2_ (19.8±1.98 ms) of the exocytotic events induced by 10 µM DMPP. Furthermore, compared with control cells, CytoD also increased foot duration (21.5±1.76 ms). These data confirm that cortical actin polymerization controls both, expansion of the initial fusion pore and catecholamine quantal release.

**Figure 6:**
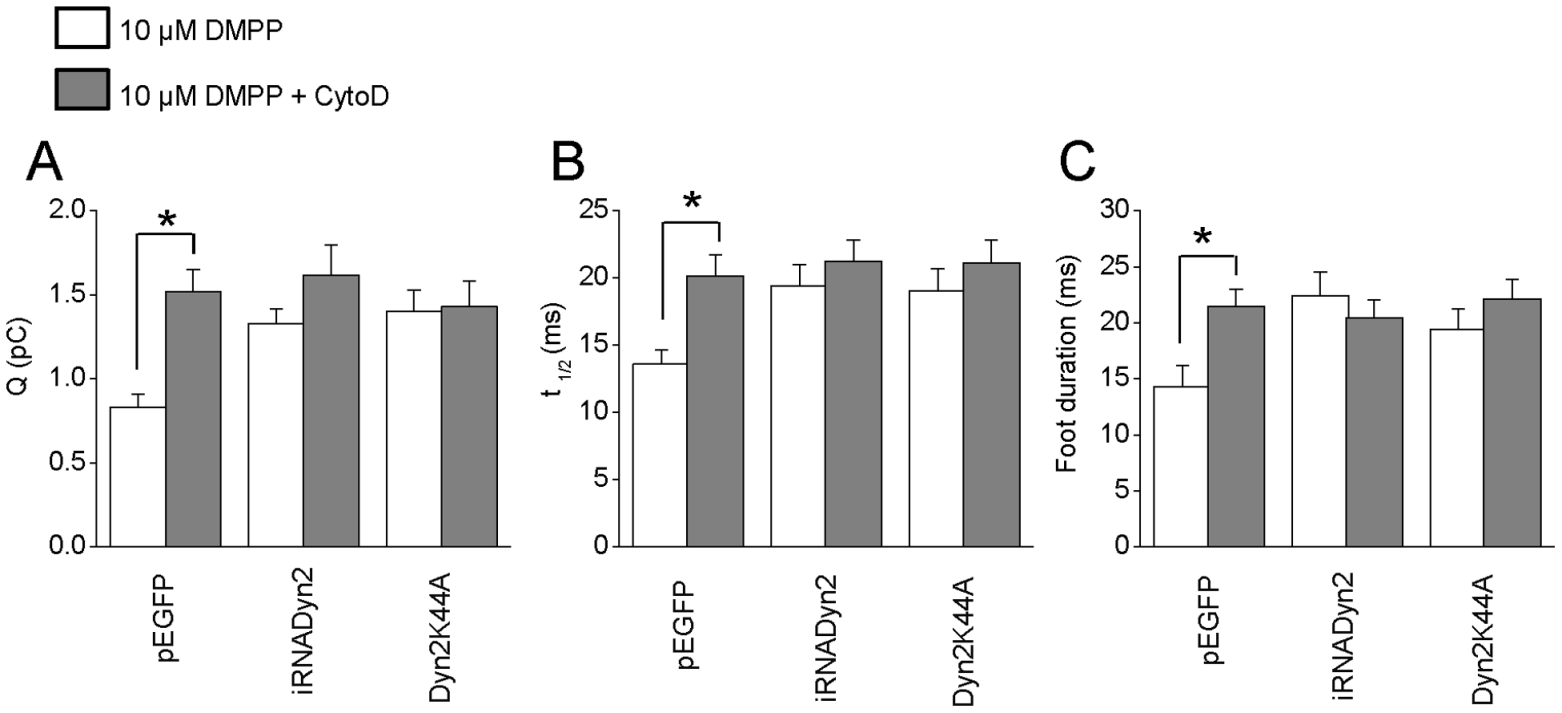
Dynamin-2 and actin polymerization regulate the fusion pore expansion and quantal size in BCC. Chromaffin cells were incubated with 4 µM CytoD during 10 minutes at 37°C. After that the exocytosis was evoked with 10 µM DMPP. **A–C:** Data show average values ± SEM of Q (A), t_1/2_ (B) and foot duration (C) of amperometric spikes induced by 10 µM DMPP in cells transfected with pEGFP (n = 27), Dyn2K44A (n = 13) or iRNADyn2 (n = 16). All amperometric parameter values correspond to the median values of the events from individual cells, which were subsequently averaged per treatment group. Thus, n correspond to the number of cells in each treatment group. Note that the CytoD treatment (grey bars) significantly increased Q, t_1/2_ and foot duration of the exocytotic events in cells transfected with pEGFP, without additional effects in cells transfected with Dyn2K44A or iRNADyn2. * p<0.05 compared with the untreated cells (Kruskal-Wallis test).

We also analyzed the effects of CytoD in cells expressing pEGFP, iRNADyn2 or Dyn2K44A. Similarly to what occurs in untransfected cells, CytoD treatment in pEGFP-transfected cells increased significantly Q (1.5±0.1 pC), t_1/2_ (20.2±1.5 ms) and foot duration (21.5±1.6 ms) of the exocytotic events (Fig. 6A–C). However, in cells transfected with iRNADyn2 or Dyn2K44A, the CytoD treatment did not induce any additional increase in Q, t_1/2_ or foot duration (Fig. 6A–C), suggesting that endogenous dynamin-2 and F-actin work through a common mechanism regulating the fusion pore expansion and the quantal release of hormones in BCCs.

## Discussion

Dynamin-2 is a widely ubiquitously expressed GTPase whose mutations cause severe hereditary neuropathies and myopathies in humans [38]. The cellular mechanisms underlying these diseases are still unclear, and do not necessarily include a dysfunction on clathrin-dependent endocytosis [39], [40], therefore, the knowledge of the spectrum of dynamin-2 functions is pivotal. In the present work we demonstrate that dynamin-2 is robustly expressed in chromaffin cells, and that it is involved not only in endocytosis but also in exocytosis and actin cytoskeleton dynamics.

The fusion pore dynamics appears to adjust the type and amount of transmitters released during exocytosis [41]. Once opened, the fusion pore can close again [34], [42], or expands and then reseals after dilation [43], thus controlling the amount of molecules being released. The ability of dynamin to generate membrane curvatures and trigger membrane fission [44] has encouraged several authors to propose that this GTPase is responsible of resealing the secretory vesicle after expansion of the fusion pore [22], [23], [27]. This should explain why dynamin disruption increases the quantal size and the duration of the exocytotic events, which is consistent with our findings and previous reports [24], [26]. Nonetheless, other explanations are also plausible. For instance, it has been described that in chromaffin cells vesicular catecholamine content changes in tandem with granule size [45], [46]. This could induce a change in both, the quantal size and foot duration [47]. Previously, we observed that acute disruption of dynamin-synaptophysin association increases the quantal size without modifications in granule volume [26] supporting the idea that dynamin does not influence the granule size. In the current work, we found no changes on the mean diameter of chromaffin granules in cells treated during 1 h with dynasore (Fig. S2), additionally discarding a role of dynamin in regulating granule size. As dynamin-2 regulates granule formation from the Golgi apparatus in neuroendocrine cells [48], we checked whether disruption of dynamin-2 function might affect the number of granules. Our analysis yielded values of 0.63±0.04 and 0.55±0.05 granules/µm^2^ for control and dynasore-treated cells respectively, demonstrating that dynasore does not affect the number of granules. However, the role of dynamin-2 on granulogenesis in BCC would require further investigations and the use of more potent inhibitors like dyngo4A [49], [50].

Another interesting possibility is that dynamin disruption favors compound exocytosis, a specialized form of secretion in which vesicles undergo fusion with each other before or during exocytosis [51], producing release events with larger quantal size. However, electron microscopy examination of 10 µM DMPP-stimulated and dynasore-treated cells did not reveal granule-granule fusion (Fig. S3). Hence, the most plausible explanation for the effects of dynamin-2 disruption on the quantal size is that this GTPase favors the closure of an already expanded fusion pore, restricting the amount of transmitter released. However, we remain puzzled on how dynamin can accelerate the expansion of the fusion pore, as observed here (Fig. 2) and by other authors (21).

Both dynamin-1 and -2 favor diverse types of fusion processes including acrosomal reaction [52], cell to cell fusion [53], [54] and fusion of virus with host cells [55], [56]. In these processes, as in exocytosis [21], dynamin appears to act facilitating the expansion of the fusion pore [52], [56]. The mechanism is still elusive but likely relies on dynamińs ability to sense curvature and remodel membranes, and probably requires the participation of other molecules. Here we propose that dynamińs action on the fusion pore expansion depends on the cortical actin dynamics, which is also involved in the aforementioned fusion processes [57]–[60].

In secretory cells, cortical actin is reorganized during the exocytotic process [18]. Moreover, as observed by us (Fig. 6) and by other authors [19], [61],actin cytoskeleton disruption with CytoD prolongs the fusion pore lifetime and increases the quantal size of the exocytotic events. These effects were not related with changes on the granule size evaluated by capacitance measurements [19] or by electron microscopy (Fig. S2). Here, we show for the first time that new actin filaments are assembled beneath the plasma membrane in a process that depends on the Ca^2+^ concentration (Fig. 4), as well as on dynamińs GTP-ase activity (Fig. 5), supporting the idea that dynamin-2 plays a pivotal role in the rearrangement of cortical actin during exocytosis.

The fact that CytoD effects on the exocytosis were no longer visible when the endogenous dynamin-2 was disrupted (Fig. 6), additionally suggests that dynamin-2 works through/or in association with F-actin in regulating both fusion pore expansion and quantal size. We have previously reported that *de novo* actin polymerization at the exocytotic sites is under the control of the GTPase Cdc42 and its effector, the nucleation promoting factor (NPF) N-WASP [36]. Interestingly, N-WASP and dynamin-2 are known to be functionally linked to regulate actin dynamics during membrane trafficking processes [62], [63]. Recently, Samasilp and co-workers observed in mouse chromaffin cells that disrupting the association of dynamin with syndapin, a synaptic partner of dynamin that binds to its proline-rich domain, limits the expansion of the fusion pore [64]. Since syndapin also modulates actin polymerization through N-WASP [65] and interacts with dynamin-2 [66], [67], it might be a regulator of the cross-talk between dynamin-2 and other actin-binding proteins.

Based on our results it is tempting to suggest a model in which dynamin-2, probably in concert with NPFs such as N-WASP, would promote a localized actin assembly at exocytosis sites during nicotinic receptor activation. As actin polymerization *per se* can generate propulsive or retractile forces to shape membranes [68], the local actin rearrangement induced by dynamin-2 may provide the force that drives the expansion of the initial fusion pore. The participation of motor proteins such myosin II on the fusion pore expansion [19], [61], [69] is probably required too. Later, F-actin and dynamin-2 in concert could generate retractile forces to constrict a slightly expanded fusion pore, favoring the resealing of the vesicle. Since, as compared with dynamin-1, dynamin-2 exhibits a significantly higher propensity to self-assembly, an enhanced catalytic activity [70], [71] and a greater sensitivity to membrane curvature [72], this isoform is likely to be a better candidate than dynamin-1 to regulate the characteristics of exocytosis in chromaffin cells, where it is robustly expressed (Fig. 1).

The mechanism proposed here could be physiologically relevant during mild stimulation for controlling the kinetics and amount of hormones released during exocytotic events.

## Methods

### Molecular Biology

Dynamin-2 wild type-GFP (Dyn2WT), dynamin-2 K44A-GFP (Dyn2K44A) and EPS15 (EΔ95/295)-GFP were kindly provided by Dr. Alexandre Benmerah (Institut Cochin, Paris). The pEGFP-iRNA vector, a bicistronic plasmid that expresses both EGFP and an iRNA targeted against the sequence of dynamin-2 (iRNA-Dyn2); and iRNA-UnR were previously described [29]. For iRNA cloning, a bovine DNA fragment encoding the sequence of dynamin-2 (GAAGAGCTGATCCCGCTGG), separated from its reverse complement by a short spacer, was annealed and cloned in the *Bgl*II and *Hin*dIII sites in front of the H1 promoter of the pEGFP-iRNA plasmid. Efficiency of iRNA-induced silencing of dynamin-2 expression was addressed by western blot analysis in Hela cells (Fig. S4).

### RT-PCR and Western-blot Assays

Total RNA from cultured bovine chromaffin and PC12 cells were prepared using the GenElute Mammalian total RNA miniprep kit (Sigma). The RNA (2 µg) was transcribed into cDNA using oligo (dT) 12–18 and SuperScriptII Reverse Transcriptase (Invitrogen). 1 µl of the cDNA was used for amplification of the PLSCR1 transcripts by PCR using Taq DNA polymerase (Sigma) and specific primers (forward primers 5′-GATATGGTAGTCAGTGAGCTCACG-3′ and 5′-GACCTGGTTATCCAGGAGCTAATCA-3′, reverse primers 5′-AACACGCTCAGGGTACACGCCA-3′ and 5′-GGTCCATGGAGAAGGTGTTCTC-3′ for dynamin-1 and -2, respectively). PCR reactions were run for 35 cycles and PCR products (605 bp for Dynamin-1 and 645 bp for dynamin-2) were analyzed on 1% agarose gel. A control without Reverse Transcriptase was performed in parallel to rule out genomic contamination.

The expression of dynamin isoforms from BCC, bovine brain tissue and from PC12 cells was evaluated by western-blotting using specific antibodies for dynamin-1 (Santa Cruz #sc-12724) and dynamin-2 (BD#610264). The respective extracts were separated by electrophoresis on a 4–12% SDS-polyacrylamide gel gradient and then proteins were electrotranferred to nitrocellulose membranes. Chemiluminescence was developed using the Super Signal West Dura Extended Duration Substrate system (Pierce). Immunoreactive bands were detected using the image acquisition system Chemi-smart 5000 and quantified using Bio-1D software (Vilber Lourmat).

### Chromaffin Cells Culture, Transfection and Amperometric Detection of Exocytosis

Bovine adrenal chromaffin cells were isolated as previously described [73] and incubated at 37°C in a 5% CO_2_. Cells were kept at 37°C at least 24 hours before the experiments. Transient transfections were performed using an Amaxa Nucleofector II Device (Lonza, Switzerland), according to the manufacturer’s instructions.

Amperometric recordings were performed as previously described [74] using carbon fibers of 5-µm diameter (Thornel P-55; Amoco Corp) and an AXOPACTH 1C-patch clamp amplifier modified according to the manufacturer instructions (Molecular Devices Corporation). The amperometric signal was low-pass filtered at 1 KHz and digitized at 5 Hz with a PCI-6030 E analogue to digital converter (National Instruments Corp.), controlled by a WinEDR software (University of Stratchclyde, UK). During recording, cells were perfused with a KREBS/HEPES solution (mM: 140 NaCl, 5.9 KCl, 1.2 MgCl2, 2 CaCl2, 10 Hepes-NaOH, pH 7.4) and exocytosis was evoked by 10 s pressure ejection of 10 µM DMPP.

### Internalization Assays

Anti-DBH antibody internalization assay was performed as previously described [32]. Briefly, 48 h after transfection with dynamin-2 constructs, cells were washed in Locke’s solution (140 mM NaCl, 4.7 mM KCl, 2.5 mM CaCl2, 1.2 mM KH2PO4, 1.2 mM MgSO4, 11 mM glucose and 15 mM HEPES, pH 7.2) and stimulated with 59 mM KCl for 30 s at 37°C. Then, the cells were incubated with a polyclonal anti-DBH antibody (1/1000) for 30 min at 4°C, washed with Locke’s solution at 37°C for 15 min, fixed and processed for immunofluorescence using an Alexa-555-conjugated secondary antibody. The distribution of DBH-containing granules was analyzed by Euclidean Distance Map [32].

### Electron Microscopy

Cultured chromaffin cells were incubated for 10 minutes at 37°C with 4 µM CytoD or for 1 hour with 100 µM dynasore, fixed for 1 h with 2.5% glutaraldehyde and 2% paraformaldehyde in 0.1 M sodium cacodylate and then rinsed and post fixed for 2 h with 1% osmium tetroxide and 0.5% potassium ferrocyanide (reducer osmium) to enhance membranes. Cells were dehydrated and embedded in Epon Resin (Embed-812, EMS). Ultrathin sections were obtained with a Reichert Ultracut-E ultramicrotome, mounted on copper grids and contrasted with uranyl acetate followed by lead citrate. Samples were observed in a Zeiss EM 900 transmission electron microscope operating at 50 kV. Images for analyses were recorded with a 7,000 X magnification. For the quantitative morphometric analysis of the chromaffin granules we included vesicles with an electron dense core and an intact vesicle membrane. Three different investigators made independent measurements from the same images and resulting vesicle sizes were averaged. The area of chromaffin granules was determined using the public domain Image J software (NIH, Bethesda, MD, USA). Granules diameter was calculated assuming a spherical shape [diameter = 2*(area/π)^0.5^ ] and corrected using a previously published algorithm [75]. The secretory granules were counted using an ImageJ macro that allows automatically select dark objects. Erroneously selected objects were manually removed to obtain a corrected number of granules.

### TIRF Microscopy to Visualize F-actin Organization in vivo

To obtain high resolution *in vivo* images of the cortical actin organization pattern we used TIRFM. Briefly, living plated chromaffin cells, transfected with Life-act-ruby or co-transfected with Life-act-ruby and GFP-dynamin-2 constructs, were incubated in a Krebs-Hepes solution, mounted in a chamber and maintained at 37°C on the stage of a Nikon inverted microscope (TE2000E) equipped with a 100X 1.49 N.A oil immersion objective, a white light TIRF and an intensilight illumination system. Images were acquired using a CCD cooled camera (Nikon Digital Sight DS-2MBWc) driven by the ACT2U 1.72 software (Nikon Instruments, Inc). The depth of the evanescence wave was estimated by the use of two independent tools: 1) 1 µm-diameter microspheres stabilized in a 1% agarose gel, and 2) a cell line transfected with a GFP tagged-membrane protein to visualize the forward transport vesicles. We determined that the maximal distance from the glass, in which the structures can be visualized, goes from 200 to 500 nm approximately. TIRF images were analyzed and processed using the Image-J software (NIH, USA).

### De novo Actin Polymerization in Permeabilized Cells

To evaluate *de novo* actin polymerization, cells were permeabilized during 6 minutes with 20 µM digitonin in KGEP buffer (mM: 139 K-glutamate, 20 PIPES, 5 EGTA, 2 ATP-Mg^2+^ and different free calcium concentration, pH 6.6) in the presence of 0.3 µM Alexa Fluor 488 G-actin conjugate (Molecular Probes) and then fixed with 4% paraformaldehyde for confocal microscopy visualization (upright Eclipse Nikon 80i). Total F-actin was stained using 1 µM phalloidin-rodhamine B. Confocal images were analyzed and processed using the Image-J software (NIH, USA).

### Data Analysis and Statistics

Amperometric spikes were analyzed using macros for IGOR (Wavemetrics) specifically designed to filter, identify and analyze individual amperometric spikes. All used macros can be downloaded from the web-site: http://webpages.ull.es/users/rborges/. The analysis was restricted to spikes with amplitudes >10 pA. For foot-signal, the analysis was restricted to spikes with foot amplitudes >1 pA and foot durations >3 ms. Data of amperometric spikes were averaged by individual cell, thus, data presented correspond to means ± SEM of cell averages from at least three different cultures. For amperometric data and image analysis “n” refers to the number of tested cells. Statistical comparisons were performed utilizing the Kruskal-Wallis test for nonparametric data or ANOVA test for parametric data. Results are expressed as mean ± standard error of the mean (SEM).

### Ethics Statement

The present work includes the use of samples from animals (bovine adrenal glands) obtained from a local slaughterhouse, Frigorific Don Pedro, certificated (Livestock role 04.2.03.0002) by the Agriculture and Livestock Service of the Chilean Government and regularly inspected by a veterinarian of the Chilean Health Service. Transport, processing and elimination of the samples were carried out in strict accordance with the Article 86 of the Sanitary Regulations of the Chilean Government (Supreme decree N° 977/96).

The protocols described in this article were approved by a Committee of Bioethics and Biosafety of the Faculty of Science, University of Valparaíso, directed by Professor Juan Carlos Espinoza, on March 7, 2011.

## Supporting Information

Figure S1 Expression of iRNADyn-2 and Dyn2K44A efficiently inhibit compensatory endocytosis of chromaffin granules.
(PDF)

Figure S2 The acute inhibition of dynamin’s GTP-ase activity or the disruption of actin dynamics does not change the size of chromaffin granules in resting cells.
(PDF)

Figure S3 Inhibition of dynamin GTP-ase activity does not induces fusion between chromaffin granules in stimulated cells.
(PDF)

Figure S4 Figure S4: iRNA-Dyn2 decreases the expression of dynamin-2.
(PDF)
