# Supplementary material for: Dynamin-2 Regulates Fusion Pore Expansion and Quantal Release through a Mechanism that Involves Actin Dynamics in Neuroendocrine Chromaffin Cells
Source: PLoS One. 2013 Aug 5;8(8):e70638. doi: 10.1371/journal.pone.0070638 (PMC3734226; doi:10.1371/journal.pone.0070638)
Supplement: Figure S1 — Expression of iRNADyn-2 and Dyn2K44A efficiently inhibit compensatory endocytosis of chromaffin granules. (PDF) [file pone.0070638.s001.pdf]

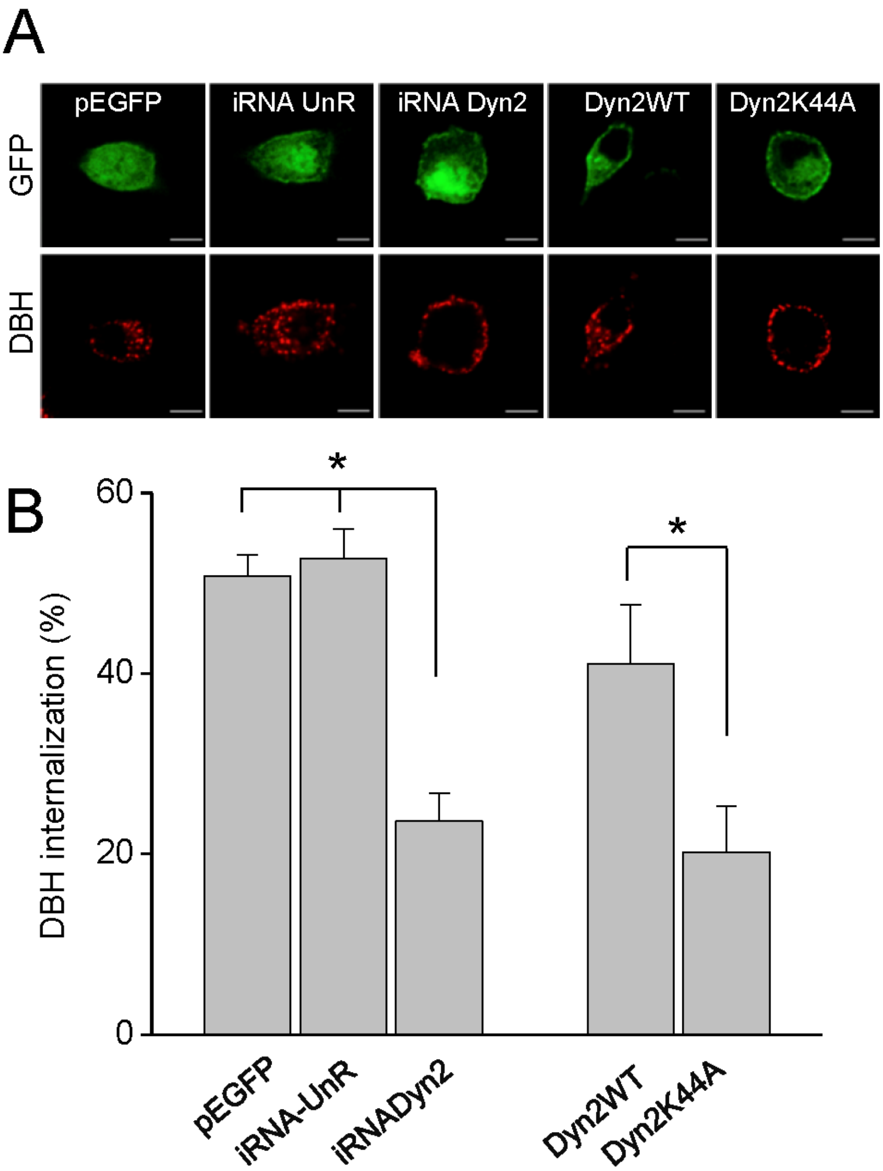

Figure S1: Expression of iRNADyn-2 and Dyn2K44A efficiently inhibit compensatory endocytosis of chromaffin granules. Chromaffin cells transfected with pEGFP, iRNA-UnR, Dyn2WT, Dyn2K44A or iRNADyn2 were stimulated with high potassium, incubated with an anti-DBH antibody and then fixed. Anti-DBH antibodies internalization was assessed. **A:** Confocal imaging showing anti-DBH internalization and GFP labeling in cells expressing the different constructs. Scale bar= 5 µm. **B**: Analysis of the internalization of DBH positive vesicles by Euclidean distance map. Data are the mean values ± SEM (n = at least 25 cells per condition). *p<0.05.
