# Supplementary material for: Dynamin-2 Regulates Fusion Pore Expansion and Quantal Release through a Mechanism that Involves Actin Dynamics in Neuroendocrine Chromaffin Cells
Source: PLoS One. 2013 Aug 5;8(8):e70638. doi: 10.1371/journal.pone.0070638 (PMC3734226; doi:10.1371/journal.pone.0070638)
Supplement: Figure S2 — The acute inhibition of dynamin’s GTP-ase activity or the disruption of actin dynamics does not change the size of chromaffin granules in resting cells. (PDF) [file pone.0070638.s002.pdf]

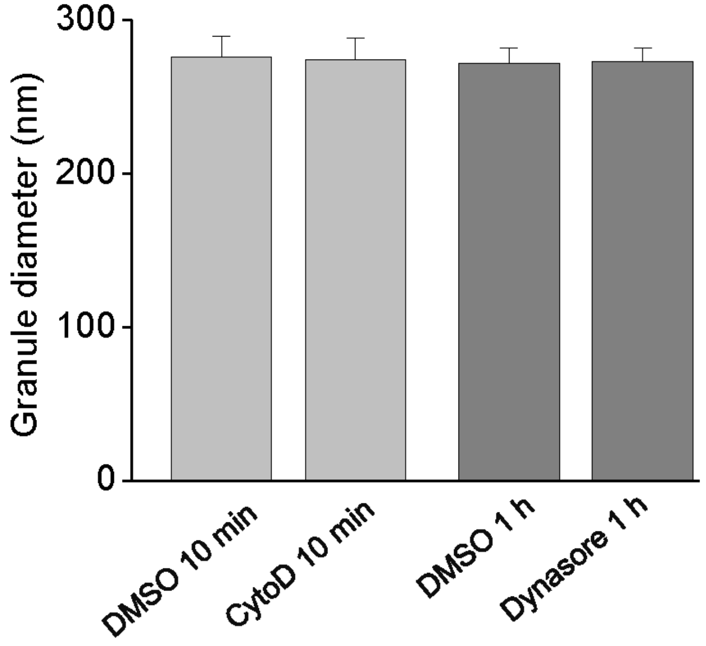

Figure S2: The acute inhibition of dynamin´s GTP-ase activity or the disruption of actin dynamics does not change the size of chromaffin granules in resting cells. Mean values for chromaffin granule diameters from bovine chromaffin cells at rest condition treated with 4 µM CytoD, 100 µM dynasore or the vehicle DMSO. At least 15 vesicles per cell and 5 cells per each condition were measured.
