# Supplementary material for: Dynamin-2 Regulates Fusion Pore Expansion and Quantal Release through a Mechanism that Involves Actin Dynamics in Neuroendocrine Chromaffin Cells
Source: PLoS One. 2013 Aug 5;8(8):e70638. doi: 10.1371/journal.pone.0070638 (PMC3734226; doi:10.1371/journal.pone.0070638)
Supplement: Figure S3 — Inhibition of dynamin GTP-ase activity does not induces fusion between chromaffin granules in stimulated cells. (PDF) [file pone.0070638.s003.pdf]

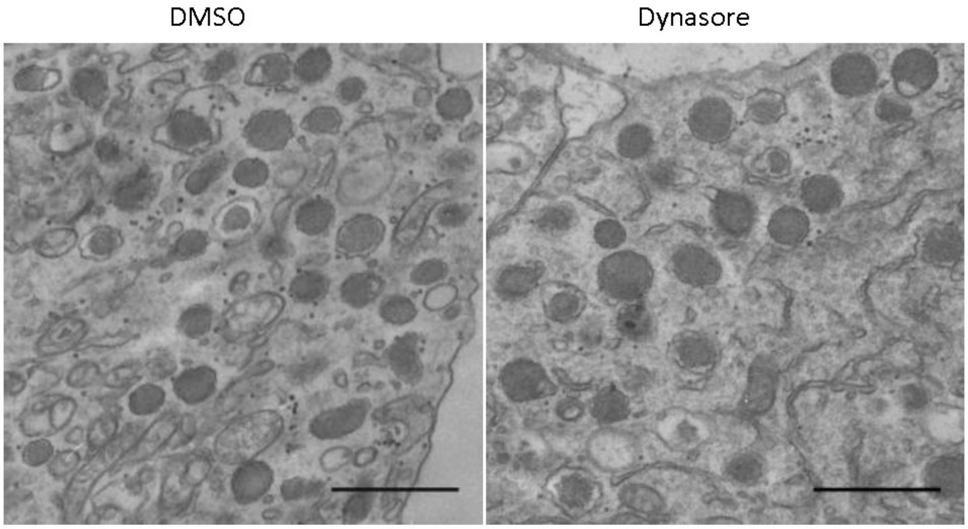

Figure S3: Inhibition of dynamin GTP-ase activity does not induces fusion between chromaffin granules in stimulated cells. No evidence of fusion between granules or compound exocytosis was observed under electron microscopy in cells stimulated with 10 μM DMPP and treated with 100 μM dynasore or the vehicle DMSO. Scale bar = 1 μm. Note that no differences in granule diameter were found between dynasore and DMSO treated cells under stimulation. At least 25 vesicles per cell and 5 cells per each condition were measured.
