# Supplementary material for: Dynamin-2 Regulates Fusion Pore Expansion and Quantal Release through a Mechanism that Involves Actin Dynamics in Neuroendocrine Chromaffin Cells
Source: PLoS One. 2013 Aug 5;8(8):e70638. doi: 10.1371/journal.pone.0070638 (PMC3734226; doi:10.1371/journal.pone.0070638)
Supplement: Figure S4 — iRNA-Dyn2 decreases the expression of dynamin-2. (PDF) [file pone.0070638.s004.pdf]

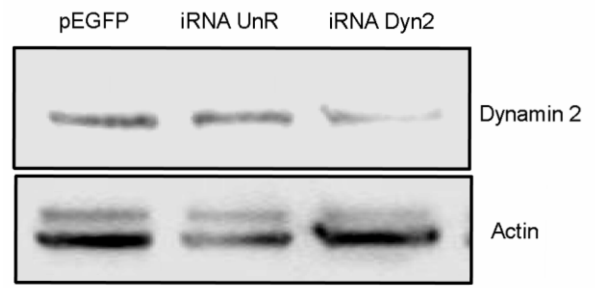

Figure S4: iRNA-Dyn2 decreases the expression of dynamin-2. As the efficiency of transfection with chromaffin cells is relatively low (less than 10%) and the siRNA sequence directed against bovine dynamin-2 has 100% similarity with human dynamin-2, we checked the efficiency of iRNADyn2 by western blot on human Hela cells. 48 h after transfection Hela cells were lysed and protein extract (100µg) were used for electrophoresis and western blot analysis using antibodies against Dynamin 2 and Actin. In this condition, dynamin-2 expression decreased by 71% as compared with the UnR-siRNA.
